# Generative AI can and should accelerate research evaluation reform to better recognize ‘distinctly human contributions’

**DOI:** 10.1093/reseval/rvag020

**Published:** 2026-04-11

**Authors:** Mohammad Hosseini, Brian D Earp, Sebastian Porsdam Mann, Kristi Holmes

**Affiliations:** Department of Preventive Medicine, Northwestern University Feinberg School of Medicine, Chicago, IL, 60611, USA; Galter Health Sciences Library and Learning Center, Northwestern University Feinberg School of Medicine, Chicago, IL, 60611, USA; Yale-Hastings Program in Ethics and Health Policy, The Hastings Center, Garrison, NY, 10524, USA; Department of Psychology, Yale University, New Haven, CT, 06510, USA; Center for Advanced Studies in Bioscience Innovation Law, University of Copenhagen, Copenhagen, DK-2300, Denmark; Department of Preventive Medicine, Northwestern University Feinberg School of Medicine, Chicago, IL, 60611, USA; Galter Health Sciences Library and Learning Center, Northwestern University Feinberg School of Medicine, Chicago, IL, 60611, USA

**Keywords:** Generative artificial intelligence, research evaluation, ethics, scholarly assessment

## Abstract

As generative artificial intelligence (GenAI) revolutionizes how research is conducted, it also challenges traditional methods of scholarly evaluation. Productivity metrics such as publication and citation counts are widely understood to be poor proxies for gauging meaningful impact. These metrics are becoming even less reliable as GenAI accelerates text-based and computational work while leaving other forms of research labor (e.g. community engagement, in-person mentorship and team development) largely unaffected. This uneven effect risks exacerbating existing evaluative biases. We argue that evaluation reforms should be organized around two categories of ‘distinctly human contributions’ that are indispensable to research, but which are inadequately captured by metrics: (1) the epistemic-ethical category, encompassing situated judgment under accountability (e.g. deciding what to trust, justifying that decision, and standing behind it); and (2) the socio-relational category, encompassing sustained forms of valuable human engagement (e.g. mentoring, teaching, community partnership and trust-building). We suggest practical mechanisms for supporting evaluation reform including modified CRediT (Contributor Role Taxonomy) statements, recognition of a broader array of outputs, and strengthened narrative CVs and third-person testimonies. However, we acknowledge that these suggestions, particularly those relying on narrative self-presentation, are themselves vulnerable to GenAI manipulation and are insufficient on their own. If distinctly human contributions to research require judgment and relationships that resist automation, then evaluation cannot be reduced to instruments designed to minimize human evaluative effort. GenAI, therefore, does not require entirely new systems of evaluation. Rather, it increases the cost of avoiding what good and ethically sound performance evaluation has always required.

## Introduction

When generative artificial intelligence (GenAI) went mainstream following the November 2022 release of OpenAI’s ChatGPT, academic researchers were among the earliest adopters. Initial reported uses were mainly ‘adjunctive’ or implementation-oriented in nature: GenAI tools were used to help refine or polish human-drafted text, write or debug code, and summarize the literature, among other ‘supportive’ activities (Nordling 2023). Today, however, researchers are increasingly using advanced GenAI-based tools (e.g. Google’s ‘co-scientist’, OpenAI’s Deep Research) for ‘core decision-making tasks,’ from brainstorming and defining research objectives to conceptualizing and designing experiments and suggesting interpretations of results in light of theory (Resnik et al. 2026).

Furthermore, when it comes to the writing of manuscripts, rather than merely suggesting edits to human-generated prose, 'frontier’ large language models (LLMs) such as Anthropic’s Claude can draft entire articles of publishable or near-publishable quality with relatively modest human input (Earp et al. 2026). In 2025, Sakana (2025) claimed to have generated the first ever autonomous LLM-driven paper to pass peer review. It seems likely that technical progress will result in further use of largely-autonomous AI systems which are, or will soon be, capable of independently executing entire research workflows from hypothesis generation to data analysis and reporting with minimal human assistance. This could result in a shift of traditional human research roles to novel, complementary roles (e.g. system architects or designers of AI-driven workflows, validators of AI-driven processing steps, and/or curators of AI outputs). Such a shift would raise critical questions about authorship, accountability, and the very definition of scholarly contribution (Resnik et al. 2026).

How can academic credit and performance evaluations keep up with this shifting landscape? After all, evaluations justify resource allocation, changes in professional standing such as tenure and promotion, and other tangible or intangible rewards (de Rijcke2023). Thus, how we answer this question carries substantial practical and ethical weight. As GenAI takes on more of the work traditionally performed by humans, it becomes necessary to calibrate our evaluation systems so that they reflect this new division of labor. In this piece, we argue that GenAI’s uneven impact across research tasks reinforces longstanding calls for evaluation reform and highlights that the reforms most worth pursuing are those that require even more time and human judgment from evaluators, not less.

## Uneven automation and productivity with GenAI

Using GenAI in research can increase speed and efficiency. Tasks that once took weeks (e.g. literature reviews, data cleaning, manuscript drafting) can be completed in hours or minutes with GenAI (though outputs always require verification, and quality varies by task and user). Nevertheless, numerous tools have improved research speed and efficiency in the past, so why should GenAI be a source of concern and motivate calibration of research evaluations?

One potential answer is that earlier tools mainly automated one or more discrete parts of the research process where inputs are converted into outputs (e.g. calculators accelerating computations, statistical software running analyses). Crucially, when using previous tools, the researcher still had to frame the problem and critically interpret the result. GenAI, by contrast, can operate across *all* research stages from suggesting inputs (brainstorming a research question or helping to formulate a questionnaire) to developing arguments, analyzing data, and producing outputs that contain relatively sophisticated interpretation, justifications, claims or arguments (Voinea et al. 2026). Furthermore, as mentioned earlier, these tools are gaining autonomy (in the sense of not technically requiring as much human oversight or intervention) in conducting research (Resnik et al. 2026). This expanded scope of activities and machine-autonomy, combined with the persuasive fluency of LLM-generated text, extends GenAI’s influence and capabilities well beyond mere efficiency gains, potentially shaping the direction, content, execution, findings, and ultimately societal impacts of the research itself (Hosseini et al. 2025).

That being said, GenAI’s impacts on research are not uniform, and this unevenness is among several factors that challenges research evaluation. Research domains differ in how much or what type of (non)automatable work precedes manuscript submission. Accordingly, domains also differ in the extent to which GenAI can speed up work without sacrificing quality. In some contexts, the path from initial research question to final publication runs largely through text, via reading, synthesizing, drafting, and revising. In these contexts, GenAI can, in principle, accelerate or automate virtually the entire process (Voinea et al. 2026). However, in practice, the quality of GenAI outputs in these domains still depends on researchers’ ability to direct the process and critically evaluate the output. For example, a senior researcher can use GenAI to accelerate the development of a publishable argument in ways that an undergraduate (who uses the same tool) cannot. This is because the expertise required to identify promising avenues, ask the right questions, prompt effectively, recognize weaknesses, and meaningfully assess GenAI output remains with the human user.

Since the potential for acceleration in text-based research is substantial, the gap widens with other contexts in which writing is only the final step after extensive preparatory labor. Such labor might include fieldwork, specimen collection, sensor calibration, sample preparation, machine operation, or essentially *embodied* observation. For example, a botanist classifying plants in a remote ecosystem or a geologist conducting on-site hazard assessments spends much of their research effort on activities GenAI cannot meaningfully accelerate (Irwin 2026). Certain writing-centric tasks (e.g. transcribing field notes and reports) may come faster with GenAI, but these remain a small fraction of the whole. The risk, then, is that performance metrics designed for a pre-GenAI era may inadvertently favor tasks where most of the substantive work happens during writing, while undervaluing contributions where most of the work happens before anyone drafts a document.

Similarly, supervisors and mentors engaged in one-on-one and in-person guidance, or community engagement experts who communicate with research participants may not experience the *same type* of acceleration in their core research tasks as those whose work is primarily computer-based. This is not to say these researchers cannot benefit, but to emphasize that the nature and scope of benefits differ because GenAI offers immediate gains for text-based workflows (e.g. manuscript drafting, literature synthesis, qualitative analysis) and can significantly accelerate certain aspects of computational work (e.g. writing and debugging code, building analytical pipelines), while offering limited assistance in tasks that rely on in-person observation, relational expertise, or culturally-specific local knowledge that does not exist in written form. Consequently, GenAI integration into research risks exacerbating evaluative biases. Researchers whose work is most amenable to GenAI integration and support may appear or become dramatically more productive under traditional metrics, while those whose contributions depend on non-automatable labor may appear less productive; not because they are less motivated or exert less meaningful effort, but because their work cannot (yet) be automated by GenAI.

## Flawed measures under increased pressure

The current evaluation system already privileges certain groups, such as those with access to resources, those affiliated with elite institutions, or those who have native fluency in dominant academic languages, above all English. Indeed, academic evaluation has long relied on easy-to-calculate metrics (e.g. h-index) to make important decisions related to hiring and promotion or resource allocation. Such metrics do have some points in their favor. For example, in mapping diverse achievements to a common ‘scale’, such metrics may enable at least rough comparisons across otherwise incommensurate disciplines, institutions, and individuals. Such metrics also, in theory, abstract away from certain biases that could color qualitative judgments (Nguyen 2026). However, this process of abstraction and simplification also comes at a cost. Many widely used metrics are poor proxies for the meaningful goals they are meant to track, including creativity, rigor, knowledge generation, and real-world impacts (Reed et al. 2021). Since metrics are easier to chase than the underlying qualities they are supposed to measure, they can incentivise researchers to pursue the metric instead of the actual goal (the so-called measurement-management problem; see, e.g. Ward 1996).

We believe that the widespread adoption of GenAI threatens to weaken already-imperfect proxies for merit. If GenAI can assistively or independently draft high-quality manuscripts in minutes or hours (Conroy 2023; Kwon 2025; Resnik et al. 2026), publication counts in fields where research does not require new data collection may rise sharply, making cross-domain comparisons of researchers or institutions based largely on raw output (albeit, often weighted by other flawed metrics, such as journal impact factor; see Bloch and Walter 2001) less meaningful than they already are. In light of GenAI’s amenability to certain tasks (mentioned above), even field-normalized metrics, such as field-weighted citation impact (FWCI; for a discussion and comparion with other metrics, see Purkayastha et al. 2019) would not be helpful because not all researchers in a certain field equally benefit from GenAI. Citation counts face a similar problem: GenAI makes it easier to assemble long lists of references. Not only may this contribute to citation inflation (Dai et al. 2021, Kim 2024, Petersen et al. 2024), but certain already-visible or highly-cited papers may be more likely to show up in AI reference recommendation systems, exacerbating the so-called Matthew Effect (i.e. unevenly distributed accumulated advantage; Wang 2014). Thus, evaluation reforms are needed more than ever, and they should capture a wider range of factors than those that can be algorithmically sped up.

Calls to expand research evaluation indicators are not new (de Rijcke2023). The Coalition for Advancing Research Assessment (CoARA, https://www.coara.org/) and the San Francisco Declaration on Research Assessment (DORA, https://sfdora.org/) have long advocated moving beyond metric-driven assessments based on publication counts, citation rates, journal impact factors, and h-indexes. But GenAI lends these calls fresh urgency.

## Toward valuing distinctly human dimensions of research

The purpose of research evaluation is to assess researchers’ merits and impacts. Since GenAI now performs a substantial and growing share of the work that evaluation has traditionally measured, a worthwhile question to ask is: How should diverse research contributions count toward evaluation given what GenAI can do? We believe that since GenAI accelerates many routine (and also increasingly complex) aspects of scholarly work, research evaluations *should place greater emphasis on contributions that remain distinctly human*. But what makes human contributions distinct? We propose two main categories:

- *Epistemic and ethical*. Research requires more than generating plausible outcomes. It requires making informed decisions grounded in knowledge and experience about which outcomes or interpretations to trust and why. Research also requires justifying interpretive decisions and, ultimately, being answerable for them. Such social and moral accountability remains a distinctive capacity of humans (Resnik et al. 2026). While GenAI can increasingly produce sophisticated analyses and even suggest theoretical interpretations, it cannot determine whether a statistically surprising result warrants revising a theoretical commitment or is better explained by idiosyncrasies in the data-collection process that only the researcher is positioned to know. Nor can it judge when a finding, though technically sound, would be misleading if published without contextualization that depends on deep familiarity with the field. Such judgments draw on accumulated disciplinary knowledge, familiarity with the specific conditions under which data were produced, and, above all, a willingness to make judgments that one is accountable for. In short, the ‘distinctly human’ epistemic and ethical contributions to research include the *exercise of situated judgments under an assumption of accountability*: the researcher must decide what to believe or defend, commit to that decision publicly, and bear the consequences if it proves to be mistaken.
- *Socio-relational*. These are sustained human engagements that research depends on but that rarely appear in widely used metrics. Consider a senior researcher who spends years guiding a junior scholar’s independent voice and supporting their career. This requires, among other things, making nuanced, contextualized, and person-specific judgments about when/how to intervene, and when to step back—a dynamic dependant on an evolving relationship that neither party fully controls. Other examples of socio-relational contributions may include a scholar whose long-term presence in a community creates relationships and commitments that shape what questions are asked and how findings are reported, or a team leader who negotiates competing interests among collaborators while maintaining a shared sense of purpose. What is striking about these relational contributions is that their value largely depends on what might, at first glance, seem to be a human ‘weakness’ or constraint compared with machines–namely, our limited cognitive and attentional capacities. We can only truly focus on one thing (or at best, a few things) at a time. In contrast to AI systems, then, whose cognitive and ‘attentional’ resources seem almost unlimited, our considered decisions about how to allocate our scarce attention, and what trade-offs and opportunity costs to accept. These considerations include what social relationships to invest in, and how to conduct them, which have a distinctive and irreducible value (Calcott and Earp 2025). Accordingly, these socio-relational aspects are not soft-skill ‘extras’ layered on top of 'real’ research, but are indispensable to many types of work. Without them, rigorous research, skills transmission, and responsible knowledge production would not be possible, because they depend on the very maintenance of these complex socio-technical systems. GenAI is not well suited to such tasks, and for the time-being, at least, these activities remain distinctly human.

These two categories are non-exhaustive and might overlap in practice, but they give evaluators something more specific and actionable than a vague list of aspirational qualities. Crucially, what is valued within each category will vary across disciplines and scope. For example, meaningful community engagement in a public health project may differ from such engagement in conservation biology; and mentorship strategies in large high-energy particle physics consortia may differ from those employed in a local digital humanities project. Any viable framework for evaluating researchers’ performance must consider these nuances.

What would such evaluation of distinctly human contributions look like in practice? Several reforms seem feasible, though, as we shall argue, none is completely new nor sufficient on its own.

1. Contribution tracking can become more granular and systematic. Modified contributorship statements based on, or adapted from, the the Contributor Role Taxonomy (CRediT) could recognize a wider array of distinctly human tasks (Hosseini et al. 2024) while also clarifying GenAI’s role in producing outputs. This way what is evaluated is not just what was produced but what human researchers, versus GenAI, actually did. Importantly, this could include such activities as designing/implementing creative and rigorous AI workflows, verifying AI systems/processes, and validating outputs/results.
2. Evaluations should recognize a wider array of outputs beyond peer-reviewed articles, including curated and reusable datasets, research software, reproducible protocols, actionable policy briefs, and educational resources, all of which reflect substantive work from a wide range of actors. Furthermore, in light of the significance of the epistemic and ethical contributions, the development of ethics oversight protocols such as those submitted to institutional review boards (IRB), institutional biosafety committees (IBC), and institutional animal care and use committees (IACUC) should be better recognized since they inform the ethical design and conduct of research (Hosseini et al. 2026).
3. Qualitative tools such as narrative CVs could allow researchers to describe contributions that quantitative metrics miss. Although several organizations have adopted them with some success (Albert et al. 2025), as we shall shortly discuss, there is an uncomfortable irony in this GenAI age when turning to narrative CVs as a corrective measure for gameable quantitative metrics.

## Discussion

### Assessing distinctly human contributions requires disctinctly human evalutions

The three suggested practical proposals are worth pursuing as scaffolding for evaluation reform. However, we recognize that they are insufficient. If distinctly human contributions to research require context-sensitive judgments that resist reduction to any formula, then the same must be true of evaluating those contributions. Assessing whether a researcher exercised sound interpretive judgment, built productive mentoring relationships, or integrated AI tools responsibly is itself an act of context-sensitive judgment, which no standardized instrument can capture, whether through a metric, a contribution taxonomy, or a narrative.

GenAI, which can produce highly convincing but misleading prose, sharpens this point: it simultaneously undermines *quantitative* proxies and renders certain *qualitative* alternatives more gameable than before. Accordingly, evaluation that aims to track irreducible judgment must itself employ irreducible judgment. In practice, this means that evaluators should engage directly with a researcher’s reasoning through reading their work closely enough to assess whether they navigated hard interpretive decisions well, not simply counting how many papers resulted. Tenure letters already support such quality determinations, when done well, but the practice is inconsistent.

### Narrative CVs can help but are not a cure

Even before GenAI, narrative formats tended to favour researchers socialized into a particular tradition of self-promotional academic prose, correlating with discipline, linguistic background, and temperament in ways that are not obviously less flawed than quantitative proxies. The mathematician who thinks in proofs should not be disadvantaged because they cannot narrate their contributions as fluently as a humanities scholar, nor should non-Anglophone researchers, whose contributions are no less significant for being expressed less smoothly in English, be disadvantaged by narrative self-promotion. Furthermore, using GenAI to write one’s narrative CV, one can spin a modest contribution into a hyperbolic and narcissistic story that sounds vaguely plausible. In other words, by encouraging the use of narrative CVs, we risk replacing one gameable instrument with another that may be even more susceptible to manipulation.

If narrative CVs are to be used, they should be strengthened by adding a section that explicitly describes the researcher’s distinctly human contributions, while also explaining how these can be independently verified. Mentoring, community engagement, and participant relationships are by definition interpersonal: their quality can only be appropriately assessed by including the people on the other side. Letters from mentees, community partners, or research participants should carry more weight than a researcher’s own account in a narrative CV (although these letters can also be written by GenAI). Mentoring outcomes, including professional and independent outcomes of junior scholars, could also provide a more concrete signal than self-descriptions of supervisory style. Documentation of community engagement and community-based partnerships should, wherever possible, come from the community itself rather than the researcher’s narrative of it.

Narrative CVs can also be further strengthened with statements about AI expertise. We believe that producing socially valuable research outcomes, such as a life-saving treatment, should count toward a researcher’s evaluation, even if the work was substantially AI-assisted. A researcher who directs AI tools toward a significant problem makes genuine contributions to research and should be allowed to discuss these in their narrative CV.

### Quantitative measures can still help

Should a researcher’s publication record in respected journals (with a well-earned reputation for rigorous peer review) be discounted in performance evaluations? Far from it. While the sheer number of publications should plausibly carry less weight than in the past (e.g. field-specific thresholds regarded as impressive should shift, and researchers ideally evaluated on a curve), turning away from such measures altogether is probably not the right response to their limitations. Instead, we believe that quantitative metrics should be better complemented by qualitative evidence about distinctly human contributions. Neither aspect should be weighed too heavily. Indeed, an overreliance on qualitative narrative evaluations or reference letters could unfairly disadvantage those who lack privileged social networks, or who may struggle with certain aspects of interpersonal relationships (e.g. due to neurodivergence), but who nevertheless make significant research contributions. On the flip side, social popularity or the ability to secure positive recommendation letters does not guarantee that one is also adding value to substantive research.

## Conclusion

We have argued that the distinctly human contributions to research lie in making context-sensitive interpretive decisions and accepting responsibility and accountability for such decisions, while also sustaining research-relevant human relationships that require presence, integrity, and trust. None of these is easily quantifiable. However, the same logic applies to evaluation. The h-index, publication counts, and citation metrics have long enabled partial avoidance of the tedious work of actually reading someone’s full research, talking to their mentees and collaborators, and recruiting independent experts in the field who can evaluate the quality and impact of their research. The practical proposals we have outlined are useful scaffolding for improving evaluations. However, nothing can fully substitute evaluators’ nuanced and context-sensitive appraisals: engaging with a researcher’s publications, weighing the testimony of those they mentored or collaborated with, and forming a considered judgment about the quality of their contributions. Research evaluation cannot rest on outcome value alone, and the distinctly human dimensions we identify could allow evaluators to distinguish among researchers whose output profiles may otherwise look similar.

This approach is expensive, slow, and resistant to scaling. But that is consistent with our central thesis. If we are serious about rewarding the distinctly human dimensions of research that cannot be easily automated, we cannot afford to evaluate these dimensions through instruments designed to minimize human evaluative effort. Such a shift can also spark new innovations and approaches to enable more efficient collection and understanding of such contributions through improved attribution models. Metrics-based criteria might usefully serve as a first step in evaluations, but there will also need to be more direct and observable assessments. For example, it will be useful to see how researchers defend their work and exercise interpretive decisions, supplemented with extensive testimony from those with whom they stand in various time-tested research relationships, both vertical (e.g. mentor-mentee) and horizontal (colleagues or collaborators, and independent peers). Such steps are needed, but are not new. Responsible evaluation has always required nuance and care. What GenAI changes most acutely, is the fundamental cost of over-reliance on weak and easily ‘gameable’ quantitative proxies.
